# Testing calpain inhibition in tumor endothelial cells: novel targetable biomarkers against glioblastoma malignancy

**DOI:** 10.3389/fonc.2024.1355202

**Published:** 2024-08-02

**Authors:** Laura Guarnaccia, Stefania Elena Navone, Laura Begani, Emanuela Barilla, Emanuele Garzia, Rolando Campanella, Monica Miozzo, Laura Fontana, Giovanni Alotta, Chiara Cordiglieri, Chiara Gaudino, Luigi Schisano, Antonella Ampollini, Laura Riboni, Marco Locatelli, Giovanni Marfia

**Affiliations:** ^1^ Laboratory of Experimental Neurosurgery and Cell Therapy, Neurosurgery Unit, Foundation IRCCS Ca’ Granda Ospedale Maggiore Policlinico, Milan, Italy; ^2^ Andremacon Srl, Milan, Italy; ^3^ Reproductive Medicine Unit, Department of Mother and Child, San Paolo Hospital Medical School, ASST Santi Paolo e Carlo, Milan, Italy; ^4^ Aerospace Medicine Institute “A. Mosso”, Italian Air Force, Milan, Italy; ^5^ Medical Genetics, Department of Health Sciences, Università Degli Studi di Milano, Milan, Italy; ^6^ Medical Genetics Unit, ASST Santi Paolo e Carlo, Milan, Italy; ^7^ Istituto Nazionale Genetica Molecolare Romeo ed Enrica Invernizzi, Milan, Italy; ^8^ Department of Neuroradiology, Azienda Ospedaliero-Universitaria Policlinico Umberto I, Rome, Italy; ^9^ Department of Medical-Surgical Physiopathology and Transplantation, University of Milan, Milan, Italy

**Keywords:** glioblastoma, angiogenesis, calpains, tumor endothelial cells, target therapy

## Abstract

**Introduction:**

Glioblastoma *IDH*-wildtype (GBM) is the most malignant brain tumor in adults, with a poor prognosis of approximately 15 months after diagnosis. Most patients suffer from a recurrence in <1 year, and this renders GBM a life-threatening challenge. Among molecular mechanisms driving GBM aggressiveness, angiogenesis mediated by GBM endothelial cells (GECs) deserves consideration as a therapeutic turning point. In this scenario, calpains, a family of ubiquitously expressed calcium-dependent cysteine proteases, emerged as promising targets to be investigated as a novel therapeutic strategy and prognostic tissue biomarkers.

**Methods:**

To explore this hypothesis, GECs were isolated from n=10 GBM biopsies and characterized phenotypically by immunofluorescence. The expression levels of calpains were evaluated by qRT-PCR and Western blot, and their association with patients’ prognosis was estimated by Pearson correlation and Kaplan–Meier survival analysis. Calpain targeting efficacy was assessed by a time- and dose-dependent proliferation curve, MTT assay for viability, caspase-3/7 activity, migration and angiogenesis *in vitro*, and gene and protein expression level modification.

**Results:**

Immunofluorescence confirmed the endothelial phenotype of our primary GECs. A significant overexpression was observed for calpain-1/2/3 (CAPN) and calpain-small-subunits-1/2 (CAPNS1), whereas calpastatin gene, the calpain natural inhibitor, was reported to be downregulated. A significant negative correlation was observed between CAPN1/CAPNS1 and patient overall survival. GEC challenging revealed that the inhibition of calpain-1 exerts the strongest proapoptotic efficacy, so GEC mortality reached the 80%, confirmed by the increased activity of caspase-3/7. Functional assays revealed a strong affection of *in vitro* migration and angiogenesis. Gene and protein expression proved a downregulation of MAPK, VEGF/VEGFRs, and Bcl-2, and an upregulation of caspases and Bax-family mediators.

**Conclusion:**

Overall, the differential expression of calpains and their correlation with patient survival suggest a novel promising target pathway, whose blockade showed encouraging results toward precision medicine strategies.

## 1 Introduction

Tumors affecting the central nervous system (CNS) are a cluster of heterogeneous neoplasia that, despite arising in a common anatomical region, differ from each other in morphology, etiology, site of onset, molecular signature, and clinical course (1). With an incidence of approximately 200,000 people worldwide every year, CNS tumors represent approximately 2% of cancer deaths (2, 3), remaining among the most difficult cancers to treat, with a 5-year overall survival (OS) <35% (4).

Among these, glioblastoma *IDH*-wildtype (herein called GBM, World Health Organization grade 4) is the most frequent and malignant glioma in adults, and the median survival rate of patients diagnosed is 15 months, with a 5-year survival rate <5%, the bottommost long-term survival rate of malignant brain tumors (5).

The difficulty encountered when fighting GBM concerns the rapid progression, immune system escape, survival, angiogenesis, invasiveness, genetic instability, high frequency of relapse, and resistance to radio and chemotherapies. Currently, the standard therapeutic strategy consists of maximal resection (when possible), followed by radiotherapy in combination with temozolomide (TMZ) (6). Despite these aggressive regimens, most patients suffer from a recurrence in <1 year and the majority succumb to the disease within 2 years of diagnosis (7). The typical invasiveness of GBM into the surrounding tissues determines a progressive deterioration of patients’ cognitive skills, significantly increasing the morbidity associated with the disease (8).

During cancer progression, especially in aggressive tumors such as GBM, neoangiogenesis closely contributes to mass expansion, through the delicate synergy between cancer stem cells, neoplastic cells, and endothelial cells (ECs). Notably, the necrotic core of GBM is characterized by an hypoxic environment, able to increase the demand for oxygen and nutrient supply to support tumor survival (9), by the activation of pro-angiogenic mechanisms.

However, the pathological unbalance between pro- and antiangiogenic mediators often generates ultra-structurally abnormal vessels, dilated, irregularly branched, convoluted, and larger than the physiological ones, with increased permeability due to the lack of a complete basal membrane and the presence of a widely distributed fenestration (10). The hyperpermeability of tumor vasculature leads to local edema and extravasation of plasma, thus increasing the interstitial pressure and altering blood flow and leukocyte flux (11, 12). The leakiness of newly formed blood vessels means that large tumor areas are not supplied with blood flow and are therefore not reachable by circulating pharmacological molecules, nutrients, and oxygen, establishing large ischemic and necrotic regions, in a self-sustaining cycle (13).

Numerous observations reported that calpains, a well-conserved family of intracellular cysteine proteinases, are strongly activated by growth factors, primarily vascular endothelial growth factor (VEGF), so that calpains residing in the ECs have a considerable impact on tumor angiogenesis (14).

Belonging to a family of calcium-dependent cysteine proteases, calpains (encoded by *CAPN* genes) proteolytically process substrates to transform their structures and modulate activities. For their pleiotropic activities, it appears reasonable that calpain family is differentially expressed in human cancers, highlighting the key role of calpains in tumor onset and advancement (15).

Some reports demonstrated that calpain expression levels in many cancer subtypes, detected by immunohistochemistry or mRNA, are directly correlated to histopathological markers of malignancy and worst clinical course, fueling the consideration of calpains as negative prognostic markers. However, it should be considered that high expression levels are not automatically associated with high proteolytic activity, as high level of calpastatin, an inhibitor efficient in binding and blocking up to four calpain heterodimers simultaneously, may counteract calpain activity (16, 17).

The scientific literature reports an altered pattern of calpain family expression, with an overexpression of CAPN1, CAPN2, and the calpain subunit 1 (CAPNS1) in several cancers, as breast cancer (18–20), colorectal cancer (21), renal cell carcinoma (22), hepatocarcinoma (23), nasopharyngeal carcinoma (24), acute myeloid leukemia (25), schwannoma and meningioma (26), and glioma (27). Conversely, high levels of calpastatin have been found in endometrial carcinomas (28). Several studies have also highlighted a direct correlation between calpain expression and clinical outcome, including response to therapy, metastatic potential, and patients’ survival (29, 30).

In the present study, we looked at assessing the involvement of calpains in GBM angiogenesis and aggressiveness, by examining GECs, the key actors of tumor vascularization and infiltration. To this aim, we evaluated i) the gene and protein expression level of calpains in GECs, ii) their association with patients’ clinical outcomes to evaluate their potential as prognostic biomarkers, and iii) the anti-tumoral and anti-angiogenic effectiveness of calpain inhibitors, with the purpose to propose a novel promising therapeutic target, as a novel approach of personalized and precision medicine.

## 2 Materials and methods

### 2.1 Study population

Patients of both sexes (n=10) with newly diagnosed GBM who underwent surgery for tumor excision at the Neurosurgery Unit of Fondazione IRCCS Ca’ Granda Ospedale Maggiore Policlinico from 2019 to 2021 were eligible for this study. The Institutional Review Board approved the protocol, and all patients provided informed consent. Inclusion criteria were as follows: 1) age between 18 and 80 years, 2) Karnofsky Performance Status (KPS) >60, 3) signed consent for the study, and 4) histologically proven diagnosis of GBM according to the WHO classification 2016 on review by two independent pathologists. Exclusion criteria were as follows: 1) previous brain surgery for other intracranial malignancies, 2) concomitant life-threatening disease, 3) history or presence of other malignancies, and 4) refusal or inability to consent to the study protocol. Demographic and clinical data covering the interval from the date of diagnosis to death or the last follow-up visit were collected from the patient’s records. Specifically, KPS, Ki-67 positivity, and MGMT promoter methylation, and IDH1/2 status were recorded.

### 2.2 Tumor sample processing and GEC isolation

Tissue samples deriving from tumor biopsies were transported from the operating room to the Laboratory of Experimental Neurosurgery and Cell Therapy under sterile conditions to be processed. From each sample, an aliquot was frozen dry at −80°C for successive molecular analysis, whereas another aliquot was manually and enzymatically digested with Trypsin (Gibco) for 1 h at 37°C. The resulting single cell suspension was plated into a 25-cm^2^ flask coated with bovine type I collagen (BD Biosciences, Milan, Italy) and cultured in endothelial proliferation medium (EndoPM) at 37°C, 5% CO_2_, 5% O_2_, according to our previous published protocols to isolate GECs (31–33). The media were changed one to two times a week and passaged at a split ratio of 1:4 every 14 days. Notably, this published and validated protocol for isolation, culture, and characterization allows us to maintain and manipulate primary GBM-derived endothelial cells (31–33).

#### 2.2.1 Immunofluorescence analysis

GECs (1×10^4^/well) were seeded into μ-Slide 8 Well, ibiTreat (Ibidi, Martinsried, Germany) collagen coated. When cells reached the desired confluence, they were fixed in paraformaldehyde 4% for 20 min at RT, washed twice with D-PBS (Euroclone) and incubated with 0.1 M glycine to quench auto-fluorescence. Then, the coverslips were incubated with PBS + 0.25%Triton ×100 to permeabilize cell membranes and then blocked in D-PBS + 5%BSA for 30 min at RT. Incubation with primary antibodies (Abs) diluted in blocking buffer was performed overnight at 4°C. The following primary Abs were used: anti-vascular endothelial growth factor (VEGF-A, Abcam), anti-Von Willebrand Factor (VWF, Sigma Aldrich), anti-VEGF receptor-1 (VEGFR1, Thermo Fisher Scientific), anti-VEGF receptor-2 (VEGFR2, Thermo Fisher Scientific), and anti-VE-Cadherin (Abcam), as specific markers for GECs. The day after, primary Abs were removed, and specific fluorescent secondary antibodies (goat anti-mouse and goat anti-rabbit, Life Technologies) were added for 45 min at RT, protected from light. After incubation and two washes with D-PBS, DAPI (1 μg/ml, Thermo Fisher) staining was performed for 3 min at RT, and coverlips were mounted with ProLong Gold Antifade Mountant (Thermo Fisher). Immunolabeling was acquired using an inverted DMI4 microscope equipped with DFC350xCCDcamera and LAS-X software (all from Leica Microsystems, Wetzlar, Germany).

### 2.3 DNA isolation

DNA from the tumor samples was obtained using DNeasy Blood & Tissue Kits Isolation kit (Qiagen, USA), following manufacturer’s instructions. DNA quantification and integrity assessment were performed with NanoDrop 3000 (Thermo Fisher).

#### 2.3.1 *IDH1* and *IDH2* mutation analysis

The assessment of hotspot mutations in IDH1, IDH2, and TERT promoter was performed as previously described (34). In brief, the molecular test investigates the following mutations in IDH1 and IDH2 genes, using MassARRAY Analyzer 4 system (Agena Bioscience, CA, USA): −IDH1, c.394C> T p.R132C, c.394C> G p.R132G, c. 394C> A p.R132S, c.395G> A p.R132H, c.395G> T p.R132L, c.395G> C p.R132P; and −IDH2, c.514A> C p.R172R, c.514A> G p.R172G, c.514A> T p.R172W, c.515G> A p.R172K, c.515G> C p.R172T, c. 515G> T p.R172M, c.516G> A p.R172R, c.516G> C p.R172S, c.516G> T p.R172S38. PCR, SAP, and IPLEX reactions were conducted as described in the manufacturer’s protocol (Agena Bioscience, San Diego, CA, USA). Samples were transferred to a SpectroCHIP (Agena Bioscience, San Diego, CA, USA) and analyzed by mass spectrometry. The spectral profiles generated by MALDI-TOF mass spectrometry were evaluated using Typer v.4.0 software (Agena Bioscience, San Diego, CA, USA).

#### 2.3.2 *MGMT* promoter methylation evaluation

The molecular test investigates the methylation of the MGMT gene promoter (GRCh37/hg19 chr10: 131.265.471–131.265.581), by pyrosequencing, with the “Pyromark Q96 ID System”. The protocol used is reported in Fontana et al. (35). DNA was extracted from FFPE tumor sample using QIAamp DNA FFPE kit (Qiagen, USA) and treated with sodium bisulfite using EZ DNA Methylation-GOLD kit (Euroclone, Italy). The test detects the methylation levels of 10 CpG sites located in the promoter region of the MGMT gene with a sensitivity of 95%. The methylation analysis was implemented as previously described (35). PCR was performed on at least 20 ng of bisulfite-treated DNA and approximately 10 pmol primers. Quantitative DNA methylation analysis was carried out on the Pyro Mark ID instrument using Pyro Gold Reagents (Qiagen) and 1 pmol of sequencing primer. Methylation data were analyzed by the Q-CpG software v1.9 (Qiagen), and the levels of methylation of each sample are represented by the mean of the methylation percentages at each CpG site of the investigated region. The analysis of the methylation levels of the MGMT gene promoter represents a prognostic and predictive marker of response to standard treatment with alkylating agents (e.g., TMZ).

### 2.4 Drug treatment

In order to assess the effectiveness of calpain-targeting in GECs, calpain inhibitor-1 (against CAPN1) and calpain inhibitor-2 (against CAPN2) were administered at 25 μM, 50 μM, and 100 μM, whereas AC-calpastatin (inhibiting both CAPN1 and CAPN2) was administered at 10 μM and 20 μM, to evaluate dose-dependent efficacy and toxicity. All compounds were purchased by Calbiochem. TMZ (Schering-Plough, Milano, Italy) 200 μM was administered to evaluate the synergistic effect of the current alkylating agent currently used in GBM therapy, with tested compounds.

#### 2.4.1 MTT assay

3-(4,5-Dimethylthiazol-2-yl)−2,5-diphenyltetrazolium bromide (MTT) assay was used to assess cell viability as a function of redox potential. GECs (5×10^3^/well) were seeded and cultured in 96-well plate for 24 h. Then, culture media were replaced with fresh media containing the specific treatments. Tests were performed in triplicate following 48 h of treatment. At the end, culture media were replaced with 100 μl of fresh media + 10 μl of MTT 5 mg/ml in D-PBS. After 4 h of incubation, media were removed, and cells were lysed with 100 μl of 2-propanol/formic acid (95:5, by vol) for 10 min. Then, absorbance was read at 570 nm in a microplate reader. Notably, the same treatments were administered to healthy brain-derived cells as astrocytes (ABM, Cat. No. T0280) and neural progenitor stem cells (NPSC, CelProgen, Cat. No. 36057-02) to evaluate if the concentrations of calpain inhibitors tested would affect normal brain cells.

#### 2.4.2 Live and dead assay

The LIVE/DEAD® Viability/Cytotoxicity Assay Kit provides a two-color fluorescence (Calcein AM and EthD-1) cell viability assay, which measures intracellular esterase activity and plasma membrane integrity, thus determining live and dead cells. GECs were seeded into 24-well plates (2×10^4^/well) until confluence and then treated with the specific treatments for 72 h. Then, the mixture of Calcein AM and EthD-1 was prepared following the manufacturer’s instruction and administered to cell cultures. Fluorescence images were acquired with an Eclipse Ti-E microscope (Nikon Instruments, Italy).

#### 2.4.3 Caspase 3/7 activity

The Caspase-Glo® 3/7 Assay is a luminescent assay added as an “add-mix-measure” format resulting in cell lysis, followed by caspase cleavage of the substrate and generation of a “glow-type” luminescent signal, produced by luciferase. GECs (5×10^3^/well) were seeded and cultured in a 96-well plate for 24 h and then treated with the specific treatments. After 72 h, Caspase-Glo® 3/7 reagent was added in equal volume, and luminescence, proportional to the amount of caspase activity, was read with a microplate reader.

#### 2.4.4 Tube formation assay

μ-Plate angiogenesis 96 wells (Ibidi) were coated with 12.5 mg/mL Matrigel (BD Bioscience), 10 μL/well on ice. After gentle agitation to ensure complete coating, plates were incubated for 30 min at 37°C to allow solidification of Matrigel. GECs (1×10^4^/well) were seeded in triplicate in EndoPM, in the presence or absence of the described treatments, and incubated at 37°C, 5% CO_2_, and 5% O_2_. Cord formation was monitored for 24 h with an inverted Eclipse Ti-E microscope (Nikon Instruments, Florence, Italy), equipped with a high-resolution cSMOS camera (Andor Zyla, Andor Technology, Belfast, UK) and NIS_Elements 4.51 software, using differential interference contrast (DIC). After 24 h of incubation, five random images were acquired and analyzed with “Angiogenesis Analyzer” plugin in ImageJ.

#### 2.4.5 Migration assay

GECs were seeded (1×10^4^ cells, each side) into Ibidi Culture Inserts (Ibidi) and cultured until 95% confluence was reached. After that, the inserts were removed, and cells were challenged with the aforementioned treatments. After 24 h, GECs were stained with 1 mg/mL Calcein AM (Thermo Fisher Scientific) for 30 min at 37°C, and images of GECs migrated into cells-free gap were acquired with an inverted Leica DMI6000B widefield microscope at ×20 magnifications in five random fields. Cells that migrated into the gap were then counted using “Analyze Particles” in ImageJ.

#### 2.4.6 Quantitative real-time PCR analysis

GECs (1×10^5^), were seeded into 25 cm^2^ collagen-coated culture flasks. When 90% confluence was reached or at the end of the above listed treatment (72h), total RNA was extracted following Invitrogen™ TRI Reagent™ (Thermo Fisher Scientific) manufacturer’s protocol and quantified with a NanoDrop 1000 spectrophotometer (Thermo Fisher Scientific). Reverse transcriptase reaction was executed using TranScriba Kit (A&A Biotechnology), loading 1 μg of RNA (A260/A280 > 1.8), according to the manufacturer’s instructions. Quantitative real-time PCR analysis (qRT-PCR) was performed using StepOnePlus™ (Thermo Fisher Scientific) using 1 μg of cDNA, forward and reverse primers (250 nM each one) and Titan HotTaq EvaGreen® qPCR Universal Mix (BioAtlas). Data were normalized to 18S expression, used as endogenous control. Relative gene expression was determined using the 2^−ΔΔCt^ method. The primer sequences are provided in **Table 1**. Notably, the gene expression level of GECs was calculated as a fold-change versus human brain microvascular endothelial cells (HBMECs, Creative Biolabs), in comparison also to endothelial cells isolated from low-grade gliomas (LGG-ECs), using the same protocol described for GECs (32).

**Table 1:** Primer sequences for qRT-PCR.

Gene	Forward primer (5′–3′)	Reverse primer (5′–3′)	Tm (°C)
*18 S*	ACTTTCGATGGTAGTCGCCGT	CCTTGGATGTGGTAGCCGTTT	61
*AKT*	TCTATGGCGCTGAGATTGTG	CTTAATGTGCCCGTCCTTGT	58
*ANG-1*	GGGCACACTCATGCATTCCT	GGTTGCACATCCAAGCCAAG	60
*ANG-2*	CCTGTTGAACCAAACAGCGG	GTGGGGTCCTTAGCTGAGTT	60
*BAX*	AGCAAACTGGTGCTCAAGG	TCTTGGATCCAGCCCAAC	57
*BCL-2*	AGTACCTGAACCGGCACCT	GCCGTACAGTTCCACAAAGG	60
*BID*	ACCGTGGTCTTTCCAGCACC	TCTGCGGAAGCTGTTGTCAG	61
*CAPN1*	CCGGCCCCTCCTCAGA	GGTCCTTGTAACCCAGGCTC	60
*CAPN2*	CCCCGACCTTTCTCTGCG	TCTCCCCAGGGATTTCGGAT	60
*CAPN3*	CGATGACCCTGATGACTCGG	CCGAAACGAAGATGATGGGC	61
*CAPNS1*	TGCGGCGCAGTGAGC	ATTGGGCCCTGGATGTTGAG	58
*CAPNS2*	GATTGTCCGCCGGTATGCTA	TTGAAGGCACGAAACATGGC	59
*CASPASE-3*	ATGGTTTGAGCCTGAGCAGA	GGCAGCATCATCCACACATAC	60
*CASPASE-7*	GAGCAGGGGGTTGAGGATTC	GTCTTTTCCGTGCTCCTCCA	61
*CAST*	ATCGCCTTCCAAACCAGGAG	TGGAGCAGCACTTCTGACTG	60
*ERK-1*	ACTCCAAAGCCCTTGACCTG	CTTCAGCCGCTCCTTAGGTA	60
*FGF-2*	TCCACCTATAATTGGTCAAAGTGGT	CATCAGTTACCAGCTCCCCC	63
*HIF-1a*	GTCTGAGGGGACAGGAGGAT	GCACCAAGCAGGTCATAGGT	61
*MEK-1*	CTTCGCAGAGCGGCTAGG	AGCTCTAGCTCCTCCAGCTT	61
*NCAM*	GCAGCGAAGAAAAGACTCTGG	ATCCTCTCCCATCTGCCCTT	60
*p21*	AGTACCCTCTCAGCTCCAGG	TGTCTGACTCCTTGTTCCGC	61
*p27*	TGGCTTGTCAGGAACTCGAC	CTAGTCTCCAGGGAGGTGCT	63
*p53*	AGGCCTTGGAACTCAAGGAT	CCCTTTTTGGACTTCAGGTG	58
*RAF*	GGTTTTGGCGTAGATTCCCC	ACCTGAAGCAAAGATGGCGT	59
*RAS*	AGCAGGTGGTCATTGATGGG	CCGTTTGATCTGCTCCCTGT	60
*TIE-2*	GGAAGGTGCCATGGACTTGA	GTCATCCTCTGTATGCCTTGCT	61
*VEGF*	TACCTCCACCATGCCAAGTG	ATGATTCTGCCCTCCTCCTTC	60
*VEGFR1*	GCAAAGCCACCAACCAGAAG	ACGTTCAGATGGTGGCCAAT	60
*VEGFR2*	CGGTCAACAAAGTCGGGAGA	CGGTCAACAAAGTCGGGAGA	60
*VWF*	ACACCTGCATTTGCCGAAAC	ATGCGGAGGTCACCTTTCAG	60

#### 2.4.7 Western blot analyses

GECs were seeded into 25-cm^2^ collagen-coated culture flasks precoated with Collagen Bovine Type I and cultured in control condition to assess basal calpain expression levels (2×10^5^), or in the presence of the desired anti-calpains treatment for 72 h (5×10^5^, each condition). In both cases, cells were lysed with M-PER Protein Extraction Reagent (Thermo Fisher Scientific) in the presence of Halt Protease Inhibitor Cocktail (Thermo Fisher Scientific) and quantified by the Pierce Detergent Compatible BCA Assay Kit (Thermo Fisher Scientific). Protein lysates (30 μg, for basal GEC expression level compared to HBMECs and 20 μg for GECs treated with calpain inhibitors) were separated in Bolt 10% Bis–Tris Plus Gels (Thermo Fisher Scientific) in Mini Gel Tank (Thermo Fisher Scientific) and transferred onto nitrocellulose iBlot 2 Transfer Stacks using iBlot 2 Dry Blotting System (Thermo Fisher Scientific). After transfer, the membrane was blocked in Tris-buffered saline/Tween20, 5% milk solution and incubated separately with anti-GAPDH, anti-calpain-1, anti-calpain-2, anti-calpain-3, anti-calpain small subunit 1, anti-calpain small subunit 2 and anti-calpastatin (all purchased from Abcam), anti-ERK1, anti-pERK1/2 (ThermoFisher Scientific), anti-Akt (Abcam), anti-MEK1 (SantaCruz Biotechnology), and anti-VEGFA (Abcam), overnight at 4°C. After incubation with HRP-labeled secondary antibody (goat-anti-rabbit IgG and goat anti-mouse IgG, Invitrogen, Carlsbad, CA, USA), protein bands were scanned with SuperSignal West Pico PLUS Chemiluminescent Substrate (Thermo Fisher Scientific) and detected by ChemiDoc XRSþ (Bio-Rad, Hercules, CA, USA). Densitometric analyses were performed using ImageJ. Notably, as for qRT-PCR, the protein expression of calpains in basal GECs was visualized and calculated as a ratio to HBMECs, in comparison also to LGG-ECs, as a lower grade brain tumor subtype.

### 2.5 Statistical analysis

All analyses were done with GraphPad Prism (GraphPad Software, Inc., La Jolla, CA, USA) and IBM SPSS (Version 29.0). Continuous variables are presented as median and interquartile range (IQR). Categorical variables are presented as counts and percentages. Parameters were compared and analyzed by a one-way analysis of variance. When significant differences were detected, Dunnet *post-hoc* comparisons versus control group were made. The Pearson correlation test and Kaplan–Meier survival analysis were performed to assess the association between calpain gene expression and patient overall survival. Data reported represent the mean±standard deviation of at least three independent experiments run in triplicate. Differences were considered statistically significant for p<0.05.

## 3 Results

The demographic, clinical, and molecular characteristics of patients, whose GBM biopsies were used to isolate GECs, are listed in **Table 2**. The median age of patients at diagnosis was 60 years (IQR, 47–76) and 67% were men. The median value of *MGMT* promoter methylation was 14 (IQR, 6.5–31.5), and all GBMs were wild type for *IDH*.

**Table 2:** Clinical and molecular data of GBM patients enrolled for GEC isolation.

	Age	Sex	Tumor location	KPS	IDH	MGMT	Ki-67
**GEC09**	81	F	FP left	60	wt	4%	15%
**GEC182**	41	F	T left	70	wt	49%	55%
**GEC183**	41	M	T right	80	wt	14%	40%
**GEC187**	45	M	FR right	100	wt	37%	20%
**GEC208**	60	M	P right	80	wt	14%	30%
**GEC210**	52	M	P left	70	wt	15%	55%
**GEC214**	66	M	T left	80	wt	60%	65%
**GEC231**	82	F	T left	70	wt	3%	27%
**GEC233**	80	M	T right	80	wt	2%	40%

M, male; F, female; T, temporal lobe; I, insular lobe; FR, frontal lobe; P, parietal lobe; KPS, Karnofsky performance status; MGMT, O6-methylguanine-DNA methyltransferase; IDH, isocitrate dehydrogenase; Ki-67, proliferation index.

**Figure 1:**
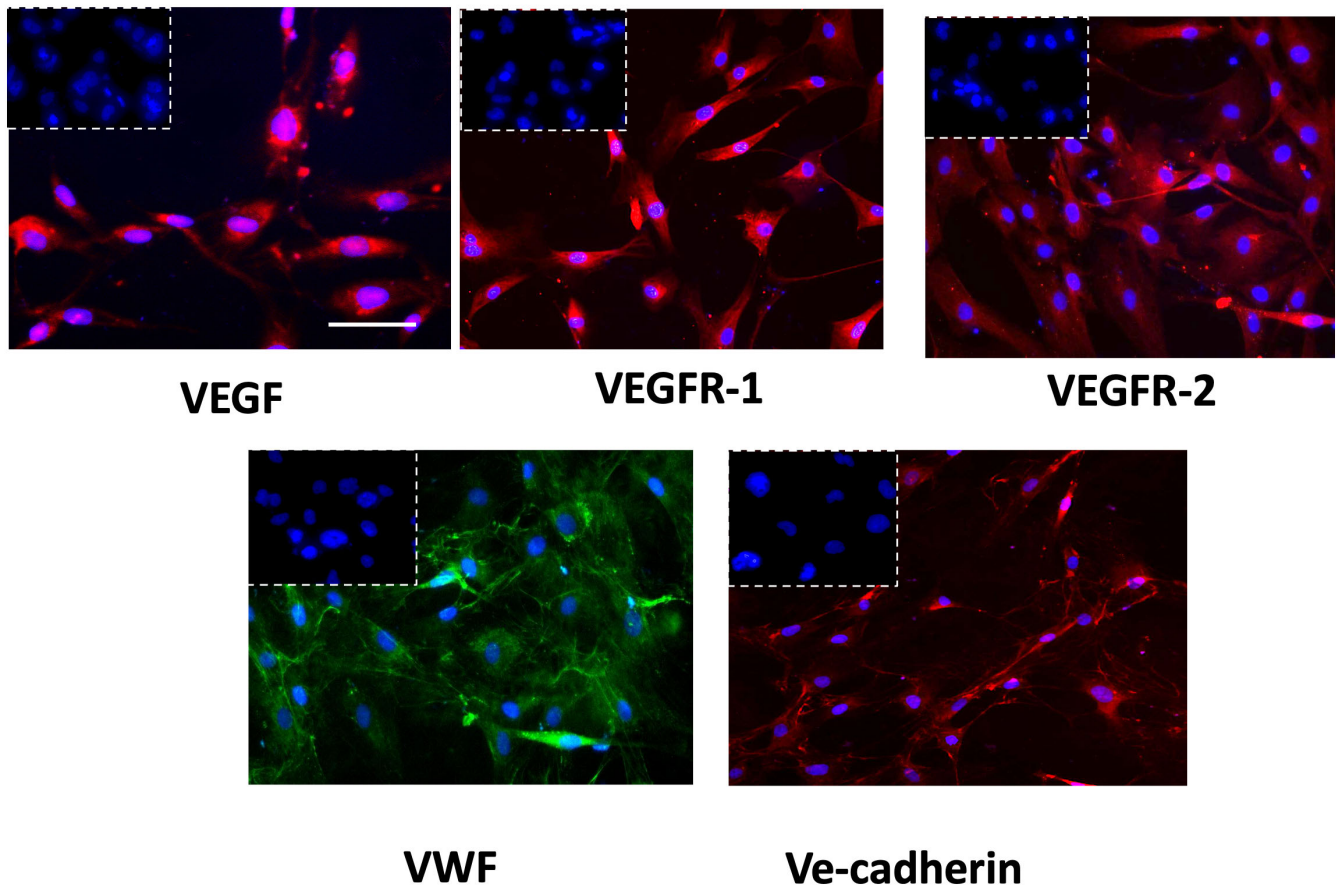
Immunophenotypic characterization of GECs by immunofluorescence for VEGF (red fluorescent labeling), VEGFR-1 (red fluorescent labeling), VEGFR-2 (red fluorescent labeling), VWF (green fluorescent labeling), and VE-Cadherin (red fluorescent labeling). Nuclei were counterstained with DAPI (blue fluorescent labeling). The white dotted boxes contain the respective isotype control for each antibody. Scale bar, 50 μm.

The successful isolation of GECs from tissue biopsies was proved by immunophenotypic characterization performed by immunofluorescence analysis. GECs displayed positivity to the principal endothelial and angiogenesis-related mediators as VEGF-A and its receptors (VEGFR1-2), von Willebrand factor (VWF) and Ve-cadherin (**Figure 1**), accordingly to our previous reports in the field (31, 32).

**Figure 2:**
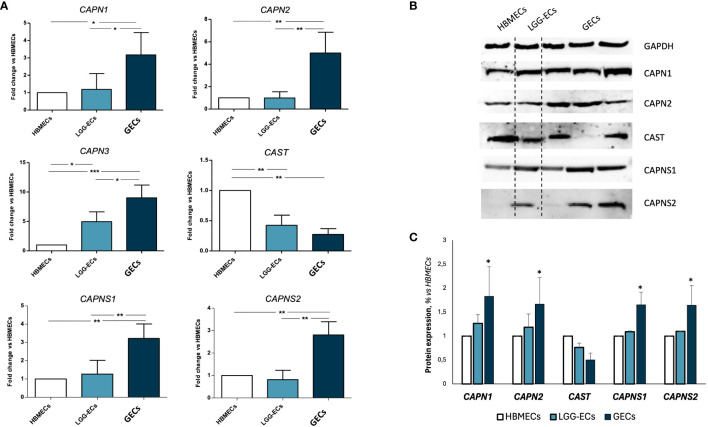
Gene and protein expression analysis of calpain 1, 2, and a3 (CAPN1, CAPN2, and CAPN3), calpastatin (CAST) and calpain small subunits 1 and 2 (CAPNS1 and CAPNS2) on human brain microvascular endothelial cells (HBMECs), low-grade glioma-derived endothelial cells (LGG-ECs), and glioblastoma endothelial cells (GECs). **(A)** Gene expression obtained by quantitative real-time PCR and reported as fold change to HBMECs, used as control. **(B)** Representative images of Western blot for calpain signaling and relative densitometric analysis **(C)** obtained by Fiji ImageJ, to quantify protein expression level, normalized to GAPDH expression level, used as endogenous control, and to untreated CTRL. Data are the mean±SD of at least three independent experiments run in triplicate. *p<0.05, **p<0.01, ***p<0.001 vs. HBMECs.

Once the GEC endothelial phenotype was confirmed, we proceeded with the gene expression screening of calpains, revealing a significant overexpression of *CAPN1-2–3*, and their small regulator subunits *CAPNS1-2*, in GECs compared to LGG-ECs and HBMECs, used as controls. An opposite trend was observed for *CAST* gene, encoding for the endogenous inhibitor of calpains (**Figure 2A**). These molecular alterations were confirmed also at the protein level, as Western blot analysis revealed a significant upregulation of calpains in GECs to the detriment of *CAST* (**Figures 2B, C**).

**Figure 3:**
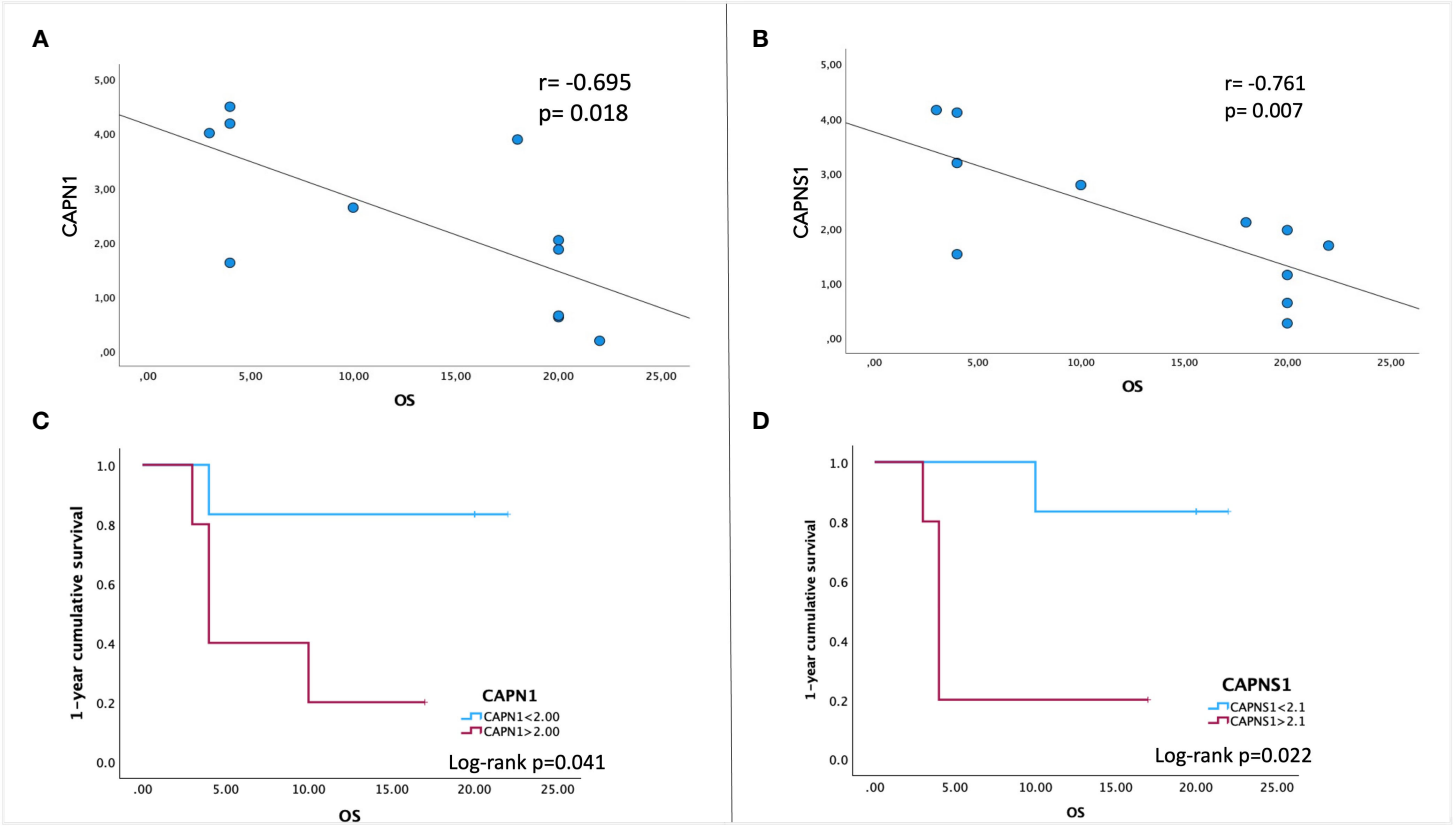
Pearson correlation between patients’ overall survival (OS) and gene expression levels of CAPN1 **(A)** and CAPNS1 **(B)**. A negative association is indicated by a negative correlation coefficient (r). **(C, D)** Kaplan–Meier survival analysis was performed to assess the association between CAPN1/CAPNS1 gene expression levels and OS. To stratify our patient cohort, the values of 2.1 for CAPN1and 2.0 for CAPNS1 (the median expression fold-change to healthy cells) were used as cutoff to evaluate the cumulative 1-year survival. Data were obtained by IBM SPSS (Version 29.0). Exact p-values are specified in the graphs.

In order to evaluate the potential prognostic value of *CAPN* expression, we performed a Pearson bivariate correlation, surprisingly revealing that, despite the small sample size, a statistically significative negative association between the gene expression level of CAPN1 and CAPNS1 and patients’ OS exists (**Figures 3A, B**). Interestingly enough, we performed a Kaplan–Meier survival analysis to assess the impact of calpain expression on OS, identifying a valid statistically significant cutoff for CAPN1 (fold change >2.1 compared to HBMECs, p=0.041) and CAPNS1 (fold change >2.0, p=0.022) as negative prognostic factors for a OS < 12 months (**Figures 3C, D**). Notably, to perform this analysis, we selected the median gene expression value of our patient cohort as potential cutoff.

**Figure 4:**
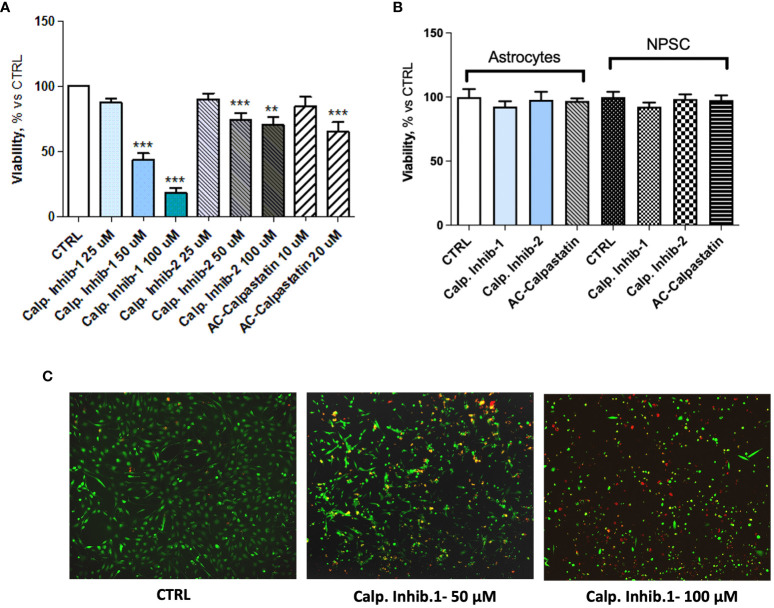
**(A)** MTT assay performed after the administration of different doses of calpain inhibitor 1–2 and calpastatin. **(B)** MTT assay performed on healthy brain cells, astrocytes, and neural progenitor stem cells (NPSC) to assess the possible toxic effect of calpain inhibitors. **(C)** Representative images of Live and Dead assay conducted to assess the toxicity of tested compounds. Data are the mean±SD of at least three independent experiments run in triplicate. **p<0.01, ***p<0.001.

These results prompted the potential to test inhibitors of calpain signaling, to curb the angiogenic process. To this aim, the effect of calpain-1 and calpain-2 inhibitors and calpastatin, the natural inhibitor of both calpain-1 and calpain-2 was assessed on GEC viability, proliferation, apoptosis, and functionality. Toxicity test was performed by MTT and Live and Dead assays with a dose escalation. These tests revealed a dose–response trend, especially for calpain-1 inhibitor, whose maximum effect was observed at the concentration of 100 μM (**Figure 4A**). Contrary, Live, and Dead assay revealed a toxic effect at this concentration, with a high percentage of necrotic cells (**Figure 4C**), suggesting carrying out further experiments using our optimal concentration of 50 μM. The doses of the other compounds were chosen accordingly. Once the optimal dose was identified, we challenged healthy brain-derived cells as astrocytes and NPSC, to evaluate the possible, but not observed (**Figure 4B**), toxic effect of calpain inhibition.

**Figure 5:**
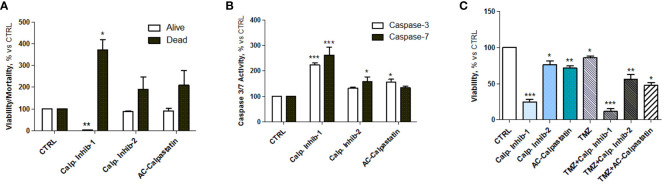
**(A)** Estimation of viable and dead GECs after treatment with calpain inhibitors 1–2 and calpastatin. **(B)** Caspase-3 and caspase-7 activity assay performed after GEC treatment. **(C)** Viability test conducted by MTT on GECs treated with calpain inhibitors 1–2 and calpastatin, alone or in combination with TMZ. Data are the mean±SD of at least three independent experiments run in triplicate. *p<0.05, **p<0.01, ***p<0.001.

The viability test on GECs showed that calpain inhibitor 1 had the strongest effect in decreasing GEC viability and promoting cell death (**Figure 5A**), as the viability reached the 20% and the mortality reached the 400% compared to untreated control. These data were confirmed by the statistically significant increased activity of caspase-3 and caspase-7, considered as effector caspases of apoptotic events (**Figure 5B**). Interestingly, the analysis of viability also revealed a synergic activity of calpain inhibitors and TMZ, the standard treatment for GBM patients, suggesting the potential efficacy of a combined treatment (**Figure 5C**).

**Figure 6:**
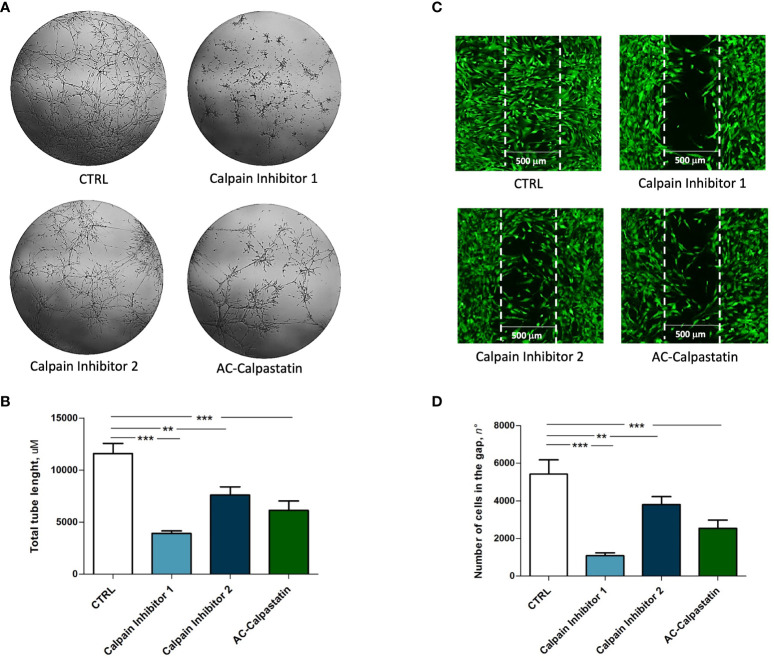
**(A)** Tube-like structure assay and **(C)** migration assay performed on GEC after treatment with calpain inhibitors. The estimation of treatment efficacy was performed by calculating the total tube length **(B)** and the number of cells migrated into the gap **(D)**. Data are the mean±SD of at least three independent experiments run in triplicate. p<0.05, **p<0.01, ***p<0.001.

Then, to test the effect of calpain inhibitors on angiogenic potential, *in vitro* tube-like structure assay on primary GECs confirmed the inhibition of vascular network formation (**Figure 6A**), with a statistically significant reduction in total tube length (**Figure 6B**). Moreover, the same effect was observed on GEC migration, as the administration of calpain inhibitors, especially calpain inhibitor-1, resulted in a significant reduction in cell migration across into the gap (**Figures 6C, D**).

**Figure 7:**
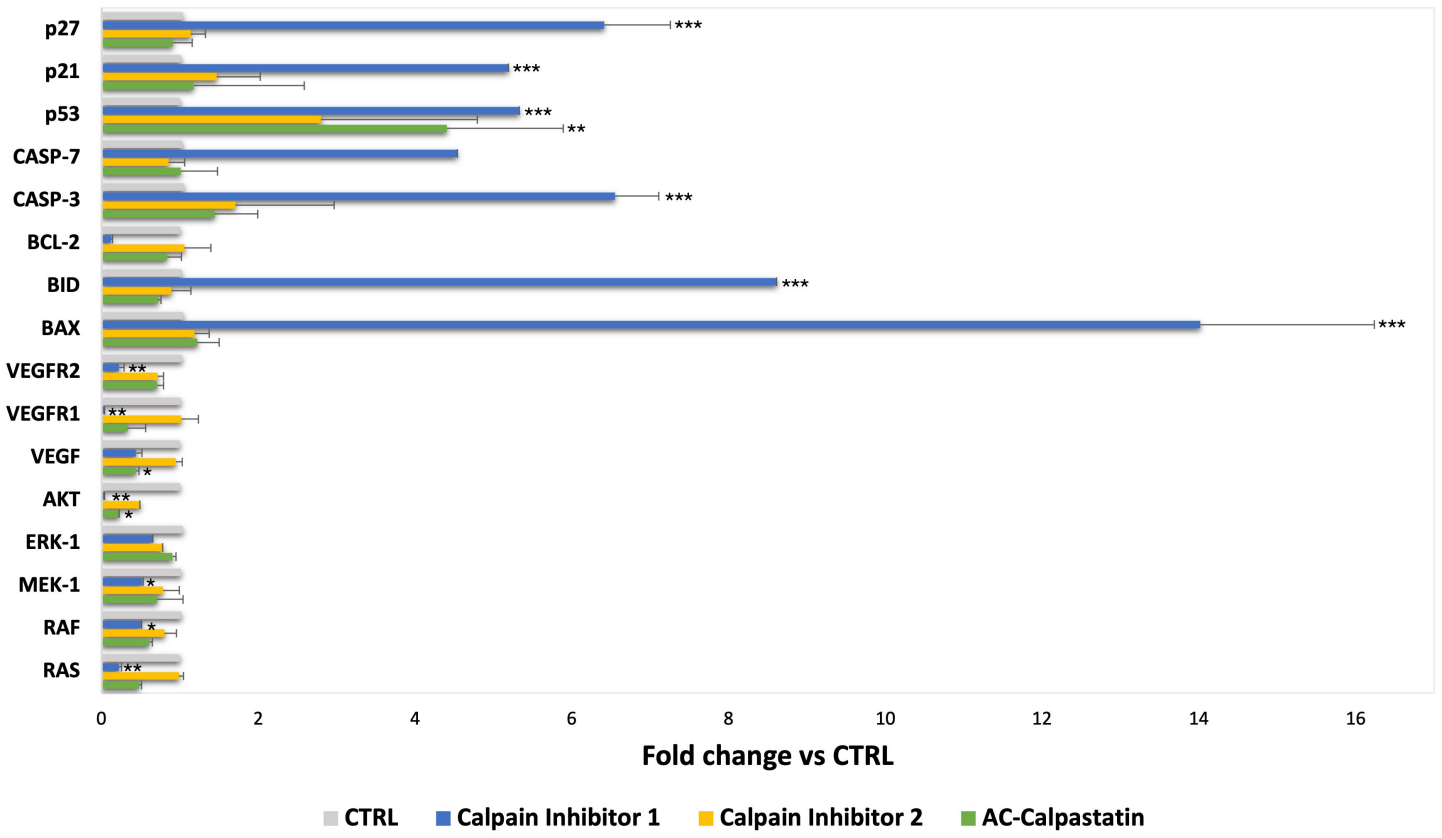
Gene expression analysis was conducted on GECs after treatment with calpain inhibitors by qRT-PCR. Data are the mean±SD of at least three independent experiments run in triplicate. *p<0.05, **p<0.01, ***<0.001.

**Figure 8:**
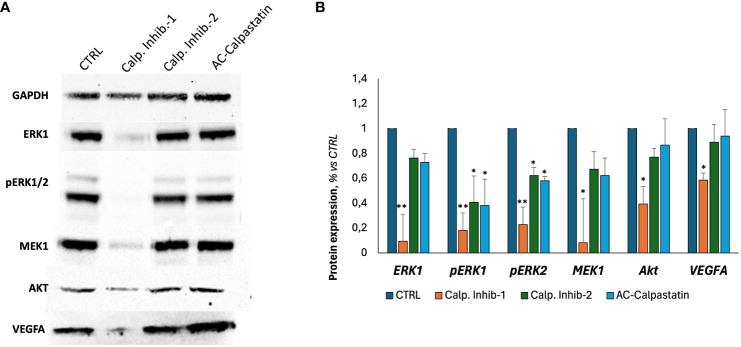
**(A)** Representative images of Western blot analyses conducted on GECs treated with calpain inhibitors for 72 h. **(B)** Densitometric quantification to assess protein expression levels of tested markers, normalized to GAPDH expression level, used as endogenous control, and to untreated CTRL. Data were obtained by Fiji ImageJ. Data are the mean±SD of at least three independent experiments run in triplicate. *p<0.05, **p<0.01.

Finally, following scientific literature and our previous studies, we explored the molecular pathways potentially impacted by calpain inhibition, through a gene (**Figure 7**) and protein (**Figures 8A, B**) expression screening. According to the previous results on GEC proliferation, survival, and functionality, calpain inhibitor 1 was found to be particularly effective in inhibiting proliferative signaling by the downregulation of proliferative signaling as MAPK, as RAF/RAS/MERK/ERK, proangiogenic pathways mediated by VEGF and its receptors and anti-apoptotic regulators as Bcl-2, and by upregulating proapoptotic mediators, as caspases and Bax-family mediators.

## 4 Discussion

GBM is the most challenging primary brain tumor in adults, resulting in the assignment of the highest grade in the WHO classification. Accounting for 54% of all gliomas, it is almost always lethal (36). Despite the aggressive therapeutic regimens, consisting of gross total resection, followed by radiotherapy with concomitant and adjuvant therapies with TMZ, the majority of patients experience a relapse during the first year after diagnosis.

The urgency to identify a novel targeted treatment able to counteract the extremely malignant behavior of GBM drove the hypothesis that a targetable molecular signature may be involved in patient’s response to treatment and, then, in patient’s survival. To verify this hypothesis, a preliminary study, whose overall results are currently the subject of bioinformatic analysis that will be reported in a manuscript in preparation, was conducted using comparative genomic hybridization (array-CGH) to identify distinctive copy number variations in patients classified as long-term survivors (LTS, OS>24 months) and short-term survivors (STS, OS<12 months).

Notably, due to short life expectancy, GBM-LTS, defined as patients who survive longer than 2 years post-diagnosis, comprise <15% of all cases; thus, comparative studies on molecular differences between LTS and STS are challenging and promising, with a potential enormous impact on clinical practice.

The analysis of chromosomic unbalances exclusive of LTS group showed the upregulation of oncosuppressor pathways, regulating cell proliferation, survival, angiogenesis, and response to treatment. The CNV investigation together with the gene ontology analysis reported in LTS patients, an enrichment of pathways related to cell activation, cell cycle process, proteolysis, calcium-dependent cysteine-type endopeptidase activity, negative regulation of apoptotic process, and positive regulation of cell survival, revealing also an altered pattern of calpain family.

Calpains represent a conserved family of cysteine proteinases able to catalyze the controlled proteolysis of many specific substrates. The biological activity of calpains influences many central cellular processes, such as proliferation, apoptosis, survival, signaling, and cytoskeleton remodeling. From these premises, we decided to examine the active involvement of calpain in GBM aggressiveness, particularly focusing on angiogenic mechanisms promoted by ECs.

Using our already developed protocols, we started isolating GECs from GBM biopsies, confirming the endothelial phenotype by immunofluorescent analysis for endothelial and angiogenesis-related markers. GECs showed positivity to the most characterized pro-angiogenic mediators, VEGF-A and its receptors VEGFR1–VEGFR2, whose signaling has been widely recognized as crucial contributor of pathogenic angiogenesis and consequent formation of abnormal, fragile, and permeable microvessels (37, 38). The action of VEGF is primarily exerted on ECs, of which it promotes proliferation, migration, and survival, so that a positive correlation between VEGF, microvascular density, and clinical outcome has been frequently reported (39, 40). Another critical pro-angiogenic factor found was Von Willebrand Factor (VWF), a multimeric plasma glycoprotein that assist platelet adhesion to EC surface and subendothelial matrix, acting also as a circulating carrier for coagulation factor VIII (41). VWF is stored within the Weibel–Palade bodies in ECs, whose activation determines its release in tumor microenvironment and blood circulation (42). This, in turn, drives platelet recruitment, aggregation, and activation, with the consequent release of pro-angiogenic platelet content, in a self-sustaining autocrine and paracrine cycle (42). Previous studies by our research team demonstrated for the first time that GBM patients presenting a preoperative VWF: Ag higher than a specifically identified cutoff experience a poorer prognosis, with a threefold higher risk of death (37).

Finally, it has been widely reported that ECs express relatively high levels of VE-cadherin (43), which is actively involved in angiogenesis, inflammation, regeneration, vasculogenesis, and tumor progression (44).

Therefore, we used GECs as a reliable model of GBM angiogenesis, and we proceeded with the examination of calpain expression in GECs, revealing an upregulation of the most active calpains, CAPN1 and CAPN2, and their small regulator subunits (CAPNS1 and CAPNS2), together with a downregulation of CAST, their endogenous inhibitor. Notably, the overexpression of CAPN1 and CAPNS1 was found to significantly correlate with patient OS, suggesting their potential to serve as novel prognostic biomarkers. Our data are consistent with those published on the Human Protein Atlas, which reports a statistically significant increase of glioma patients’ survival associated with a lower gene expression in a cohort of n=153 patients (p=0.048).

Calpains residing in the ECs are key participants of tumor angiogenesis. Their levels are induced by factors, primarily VEGF, whose axis VEGF/VEGFR2 has been found to stimulate calpain-2 dependent activation of PI3K/AMPK/Akt/eNOS pathway and consequent nitric oxide production and physiological angiogenesis (14). Hypoxic conditions characterizing aggressive tumors as GBM represent a further enhancer of calpain contribution in this pro-angiogenic self-vicious cycle.

The rapid progression of GBM necessitates a huge amount of oxygen and nutrients, supplied by angiogenic processes and pro-angiogenic factor secretion. The abundance and the key role of VEGF in this mechanism have been widely discussed, but it has been reported that VEGF in ECs is effective in activating calpain-2, so the administration of calpain inhibitors or siRNA may abolish VEGF-induced endothelial NO production and therefore angiogenesis (45–48). Furthermore, the faster growth of tumor cells due to hypoxic conditions leads to the upregulation of calpain expression and activity in ECs (49–51). Interestingly, calpain in tumor cells serves as a newly identified regulator of the hypoxia-inducible-factor (HIF-l α)/VEGF pathway (52). In particular, HIF-1 is known to induce transcription of more than 60 genes, including VEGF and erythropoietin, which assist in promoting and increasing oxygen delivery to hypoxic regions, thus promoting tumor progression.

For example, Zheng et al. have shown that hypoxia promotes calpain-induced filamin-A proteolysis in melanoma cells, which in turn facilitates HIF-1α nuclear translocation. In a tumor xenograft model, it has been observed that since VEGF is transcriptionally activated by HIF-1α, the overexpression of filamin-A is able to increase HIF-1α recruitment to VEGF promoter, thus promoting tumor angiogenesis. However, calpeptin inhibition of calpain attenuates HIF-1α nuclear accumulation and transactivation (52).

In order to investigate the involvement of calpains in tumor angiogenesis, Miyazaki et al. examined tumors and surrounding normal tissues from patients suffering from malignant astrocytoma, colon, and lung adenocarcinomas. The analysis of calpastatin immunostaining proved a significant loss of expression in tumor ECs compared to normal vessels. Furthermore, using mice harboring EC-specific transgene of calpastatin, the same authors observed a weakening of tumor angiogenesis in a Lewis lung carcinoma allograft transplantation model, potentially mediated by the inhibition of VEGF-C production through calpain/SOCS3/STAT3 (53).

**Figure 9:**
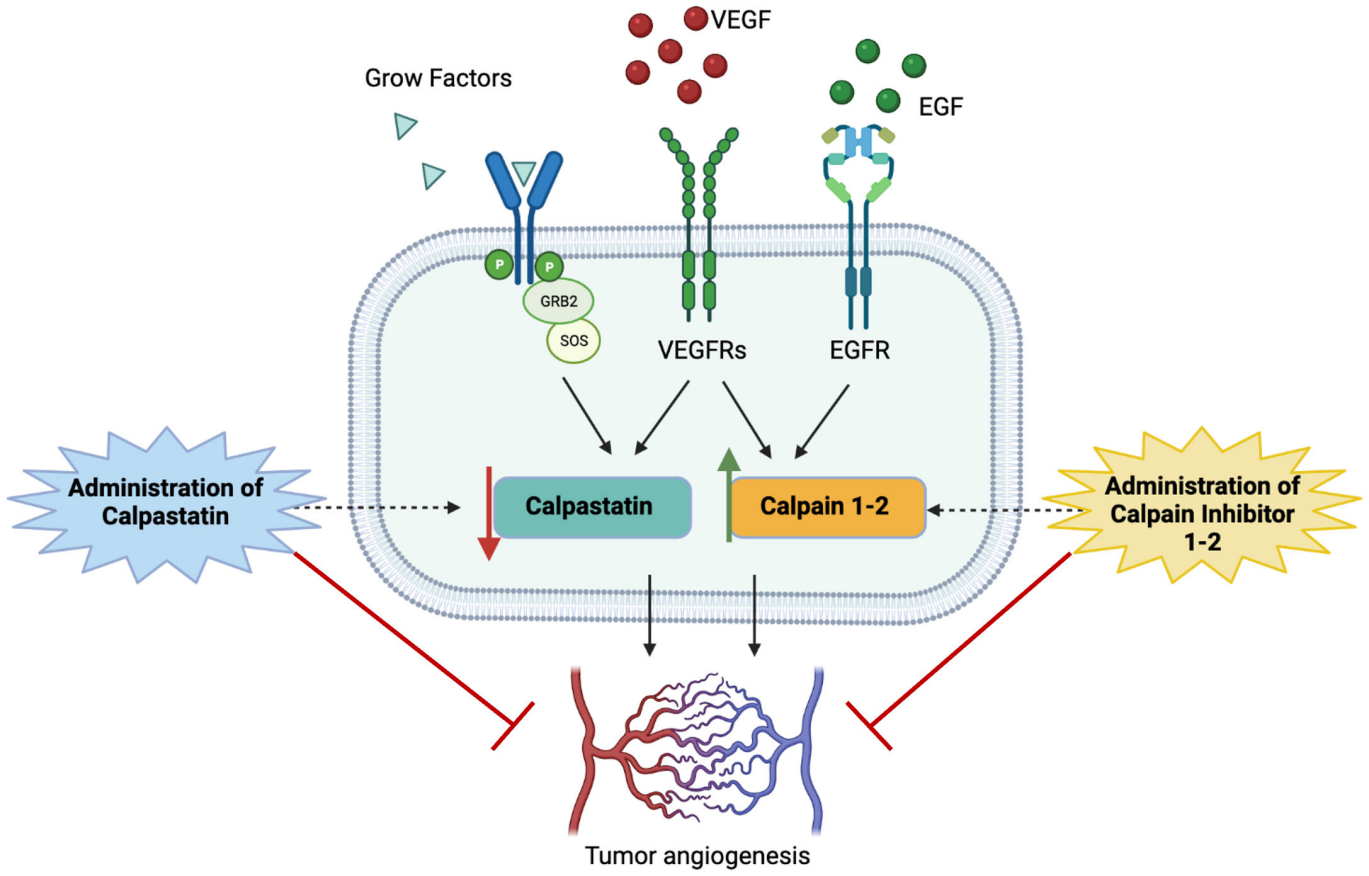
Schematic representation of study hypothesis: the hyperactivation of calpain signaling, mainly mediated by the binding of proliferative growth factors to their membrane receptors, contributes to aberrant GBM angiogenesis. Our data, consistent with scientific literature, suggest that the administration of calpain inhibitors, especially calpain inhibitor-1, may impair angiogenic mechanisms overcoming tumor invasiveness, progression, and infiltrative behavior.

Following this promising evidence about the potential pro-angiogenic role of calpains, we decided to test calpain inhibitors in order to restore the physiological condition and overcome GBM angiogenesis. To this aim, calpain inhibitors 1–2 and calpastatin were administered to GECs (**Figure 9**).

Our data demonstrated for the first time on primary GBM-derived endothelial cells, a great ability of calpain inhibitors and calpastatin to slow down GEC proliferation and survival, by inducing also apoptotic mechanisms mediated by caspase-3 and caspase-7 activation. Notably, calpain inhibitors succeeded also in inhibiting GEC functionality, as migration and angiogenesis *in vitro*. The most robust effect was observed after the administration of calpain inhibitor 1, which was able to strongly arrest cell proliferation and viability and counteract tube-like structure formation and cell migration. Of relevance, the investigation of the potential molecular mechanisms underlying these effects revealed a downregulation of MAPK and an upregulation of pro-apoptotic mediators as BAX family. The Ras/RAF/MEK/ERK (MAPK) signaling represents one of the best-characterized pathways in cancer biology, and its hyperactivation is involved in over 40% of human cancer cases. The MAPK signaling acts by switching on proliferative genes controlling cellular overgrowth and simultaneously enables cells to overcome metabolic stress by inhibiting AMPK signaling. Mechanistically, upon binding of RTKs or other stimulations, Ras small GTPases are activated by GTP/GDP exchange factors (GEFs), which in turn recruit RAF/MEK complexes to the plasma membrane and trigger the RAF/MEK/ERK kinase cascade (54).

The translocation of active ERKs into the nuclei or in the cytoplasm induces the phosphorylation of substrates implicated in cell functions, such as proliferation and survival (55–57). Hence, the aberrant activation of MAPK signaling frequently induces proliferative disorders as human cancers, as what happens in GBM (58, 59). In our data, the downregulation of MAPK is complemented by the Bcl-2 reduced expression, which impacts cell death mechanisms, including apoptosis, autophagy, and necrosis, thus operating as nodal points at the junction of multiple crucial pathways in oncology. The overexpression of Bcl-2 family proteins causes the inhibition of cell death induced by hypoxia, growth factor deprivation, and oxidative stress, so the effect of calpain inhibitors in decreasing Bcl-2 may explain the induction of apoptosis (60). These results have been further confirmed by the overexpression of Bax and Bid, the pro-apoptotic members of Bcl-2 family, and those of caspase-3 and caspase-7, the major executioner caspases of apoptosis mechanisms. Irrespective of the specific death-initiating stimulus, caspase-3 and caspase-7 are both universally activated during apoptosis, coordinating the demolition phase of apoptosis by cleaving a diverse subset of protein substrates (61). Interesting data arise also from the upregulation of p53, p21, and p27 by calpain inhibitors. The *TP53* gene encodes a protein acting as a transcription factor crucial for carcinogenesis. The inactivating mutation of TP53 is frequently detected in human cancers. The role of p53 consists in tumor suppression in response to cellular stress. The presence of the heritable TP53 mutant allele is responsible for the Li–Fraumeni disease, which predisposes patients to the development of different types of malignant tumors (62). With a similar mechanism, both p21and p27 inhibit cell cycle acting as an anti-proliferative mediators, so their deregulation, accelerated degradation, or mislocalization are often found in many cancers (63). Finally, calpain inhibitors were able to interfere with VEGF signaling, downregulating its expression and that of its two major receptors, VEGFR1 and VEGFR2, suggesting the disruption of the most active pro-angiogenic axis.

Most of the scientific literature on the relation between calpains and cancer are focused on the correlation between their expression and patient prognosis. The limited number of reports investigating their blockade are still controversial, as although there are numerous protumoral pathways induced by calpains, they are able to sensitize cancer cell to chemotherapy (18). According to our results, it was observed that calpain inhibition with calpeptin, and with a synthetic calpain inhibitor (ALLN), was able to suppress cell cycle progression and proliferation of cancer transformed cells and their anchorage-independent growth. Similarly, the inhibition of calpain activity by different inhibitors was effective in repressing the effects of the transformation induced by other oncoproteins such as v-Jun, v-Myc, v12k-Ras, and v-Fos (64). Furthermore, it has been reported that the simultaneous inhibition of calpains and ERK/MAPK pathway coupled with an activation of p38 MAPK was sufficient to restore the ability of v-Src-transformed myoblasts to differentiate (65). Interestingly, calpain inhibition was also shown to induce apoptosis of transformed cells, thanks to an accumulation of c-Myc, previously identified as a calpain substrate (66).

Furthermore, calpain inhibition, with calpeptin for example, proved to reduce lung cancer cell invasiveness by impeding cancer cell migration (67). Interestingly, the addition of C2-ceramide activating the phosphatase PP2A induces calpain dephosphorylation and inactivation, with a functional impairment of tumor invasion (68). In addition, the inhibition of m-calpain using calpain inhibitor I reduces the invasiveness of prostate carcinoma cells (69). Very similar results were obtained with rhabdomyosarcoma treated with calpeptin. Indeed, the invasiveness of these cells was dramatically reduced in the presence of calpeptin, restoring a condition close to normal myoblasts (70). Of relevance, the inhibition of calpains can also diminish the expression of metalloproteinases (MMPs). Indeed, the treatment of leukemic cells with the specific inhibitor CP1B, derived from calpastatin, affects the expression and secretion of MMP-2/MMP-9, reducing matrix degradation and thus tumor invasion (71).

Overall, the activity of calpain-1 and calpain-2, the two ubiquitous calpains, could be impaired by targeted treatment to impede cell transformation, suppress the enhanced motility, adhesion disassembly and the cell cycle progression, and by inducing tumor cell death. Interestingly, it has been reported that the inhibition of calpain activity could also be useful to improve the sensitivity of lung cancer cells to the proteasome inhibitor bortezomib (72). It is possible that while calpain inhibition may decrease apoptosis, the cells may be redirected to other modes of death. Still, the sum of the published data suggests that an interesting approach would be to target calpains to improve chemotherapy efficiency.

Furthermore, targeting calpain activity with specific inhibitors could be a novel approach to limiting the development of primary tumors and the formation of metastases, by inhibiting tumor cell migration and invasion, which allows dissemination and tumor neovascularization, which in turn allows tumor progression.

## 5 Conclusions

Taken together, our results led to the awareness that molecular mechanisms underlying GBM malignancy and aggressiveness need to be investigated deeper. Targeting calpains may be considered as a novel frontier of molecular target therapies, which may benefit from the molecular screening and consequent patient stratification. The development of target therapies for patients with brain cancer, through the modulation of angiogenesis, invasiveness, and pharmacological sensitivity/resistance, is urgently needed in the era of precision medicine. Furthermore, the discovery of novel molecular mediators, from genetics to epigenetics and proteomics, as potential prognostic and predictive biomarkers may be handy and recognizable by an “omic” approach and may impact clinical practice in terms of patient management.

## Data availability statement

The raw data supporting the conclusions of this article will be made available by the authors, without undue reservation.

## Ethics statement

The Institutional Review Board of Fondazione IRCCS Ca’ Granda Ospedale Maggiore Policlinico approved the protocol (IRB#1670/2015) and all patients provided a signed informed consent. The studies were conducted in accordance with the local legislation and institutional requirements. The participants provided their written informed consent to participate in this study.

## Author contributions

LG: Conceptualization, Data curation, Formal analysis, Funding acquisition, Investigation, Methodology, Project administration, Validation, Visualization, Writing – original draft. SN: Conceptualization, Data curation, Formal analysis, Funding acquisition, Investigation, Methodology, Project administration, Validation, Visualization, Writing – original draft. LB: Data curation, Investigation, Methodology, Validation, Writing – review & editing. EB: Formal analysis, Supervision, Validation, Writing – review & editing. EG: Supervision, Validation, Writing – review & editing. RC: Data curation, Formal analysis, Investigation, Supervision, Validation, Writing – review & editing. MM: Investigation, Supervision, Writing – review & editing. LF: Supervision, Validation, Writing – review & editing. GA: Formal analysis, Supervision, Validation, Writing – review & editing. CC: Data curation, Methodology, Supervision, Formal analysis, Validation, Visualization, Software, Writing – review & editing. CG: Data curation, Supervision, Formal analysis, Validation, Writing – review & editing. LS: Resources, Validation, Writing – review & editing. AA: Resources, Validation, Writing – review & editing. LR: Formal analysis, Investigation, Supervision, Validation, Writing – review & editing. ML: Funding acquisition, Project administration, Resources, Supervision, Writing – review & editing. GM: Conceptualization, Data curation, Funding acquisition, Investigation, Project administration, Resources, Supervision, Validation, Writing – review & editing.
